# Definition of an Normal Tissue Complication Probability Model for the Inner Ear in Definitive Radiochemotherapy of Nasopharynx Carcinoma

**DOI:** 10.3390/cancers14143422

**Published:** 2022-07-14

**Authors:** Leonie Peuker, Daniel Rolf, Michael Oertel, Alexander Peuker, Sergiu Scobioala, Dominik Hering, Claudia Rudack, Uwe Haverkamp, Hans Theodor Eich

**Affiliations:** 1 Department of Radiation Oncology, University Hospital of Münster, 48149 Münster, Germany; leo@peuker.name (L.P.); daniel.rolf@ukmuenster.de (D.R.); michael.oertel@ukmuenster.de (M.O.); sergiu.scobioala@ukmuenster.de (S.S.); dominik.hering@ukmuenster.de (D.H.); uwe.haverkamp@ukmuenster.de (U.H.); 2 Institute of Flight Guidance, Technische Universität Braunschweig, 38106 Braunschweig, Germany; a.peuker@tu-braunschweig.de; 3 Department of Otorhinolaryngology-Head and Neck Surgery, University Hospital of Münster, 48149 Münster, Germany; claudia.rudack@ukmuenster.de

**Keywords:** head and neck tumor, nasopharyngeal carcinoma, radiotherapy, NTCP, toxicity, survivorship

## Abstract

**Simple Summary:**

Definitive radiochemotherapy is the treatment of choice for locally advanced nasopharyngeal carcinoma. Due to the vicinity of the nasopharynx to the inner ear and the use of ototoxic platinum-based chemotherapy, there is a risk for irreversible damage to the auditory system. To avoid or minimize these critical side effects, radiation exposure to each inner ear must be balanced between target volume coverage and toxicity. However, normal tissue complication probability (NTCP) models of the inner ear validated by clinical data are rare. In this retrospective study of 46 patients, an NTCP model and a cutoff dose logistic regression model (CDLR) were created. There is a sigmoidal relation between radiation dose and incidence of inner ear toxicities. Dose constraints for the inner ear of <44 Gy (Dmean) or <58 Gy (Dmax) are suggested to limit the probability of inner ear toxicity < 25%.

**Abstract:**

Background: Definitive radiochemotherapy is the treatment of choice for locally advanced nasopharyngeal carcinoma. Due to the vicinity of the nasopharynx to the inner ear and the use of ototoxic platinum-based chemotherapy, there is a risk for irreversible damage to the auditory system. To avoid or minimize these critical side effects, radiation exposure to each inner ear must be balanced between target volume coverage and toxicity. However, normal tissue complication probability (NTCP) models of the inner ear validated by clinical data are rare. Patients and Methods: This retrospective study investigates the inner ear toxicity of 46 patients who received radio(chemo-)therapy for nasopharyngeal carcinoma at our institution from 2004 to 2021 according to CTCAE 5.0 criteria. For each inner ear, the mean (Dmean) and maximum (Dmax) dose in Gray (Gy) was evaluated and correlated with clinical toxicity data. Based on the data, an NTCP model and a cutoff dose logistic regression model (CDLR) were created. Results: In 11 patients (23.9%) hearing impairment and/or tinnitus was observed as a possible therapy-associated toxicity. Dmean was between 15–60 Gy, whereas Dmax was between 30–75 Gy. There was a dose-dependent, sigmoidal relation between inner ear dose and toxicity. A Dmean of 44 Gy and 65 Gy was associated with inner ear damage in 25% and 50% of patients, respectively. The maximum curve slope (*m*) was found at 50% and is m=0.013. The Dmax values showed a 25% and 50% complication probability at 58 Gy and 69 Gy, respectively, and a maximum slope of the sigmoid curve at 50% with m=0.025. Conclusion: There is a sigmoidal relation between radiation dose and incidence of inner ear toxicities. Dose constraints for the inner ear of <44 Gy (Dmean) or <58 Gy (Dmax) are suggested to limit the probability of inner ear toxicity <25%.

## 1. Introduction

Nasopharyngeal carcinoma (NPC) is a head and neck tumor entity with geographic heterogeneity occurring frequently in certain regions, such as Alaska, North Afrika, South China and Southeast Asia. Beyond the endemic regions, this tumor entity is rare with an incidence of 1 per 100,000 inhabitants per year [1,2].

The current standard treatment for nasopharyngeal carcinoma is concomitant radiochemotherapy (RCT) with intensity-modulated radiotherapy (IMRT) [3].

Anamotically, the inner ears are located close to the nasopharynx and are therefore endangered to suffer from therapy-associated (long-term) side effects. Due to the described proximity, a surgical resection is not considered to be a feasible option in most cases [4]. Hearing disorders and tinnitus are frequent late effects due to exposure to radiation doses and ototoxic chemotherapy containing platinum agents [5,6]. This demands for a careful consideration between therapeutic efficacy and toxicity profile. Burman et al. were one of the first to investigate the relation between radiation dose and complication probability, but limited their report to the middle ear without providing information on the inner ear [7]. A suggested recent dose constraint for the inner ear according to Lee et al. is a mean dose <32Gy causing a tinnitus risk <20% [8].

However, normal tissue complication probability (NTCP) models enabling risk calculations and correlation with clinical data are still rare for the inner ear, although they could be established in other organs [8,9]. This paucity of evidence prompted our group to investigate dose-side effect relationships in a collective of patients with nasopharyngeal carcinoma treated at our institution in the last 17-years. The analysis describes the occurrence of inner ear toxicities and derives a fitting NTCP model. This model is a feasible instrument to evaluate individual patient’s risk profile, counsel patients accordingly and to consider RT plan modifications in high-risk patients.

## 2. Patients and Methods

### 2.1. Study Design and Data Collection

The study was designed as a retrospective monocentric analysis including 46 patients with histologically confirmed nasopharyngeal carcinoma treated with radiotherapy or radiochemotherapy at our department between 2004 and 2021. Clinical data was collected via the electronic patient file as provided by our hospital information system (Orbis, Agfa Healthcare, Mortsel, Belgium) including medical reports, laboratory values, imaging and follow-up notes. Additional data on radiotherapy details were provided by the information system of the department of radiation oncology (Aria, Varian Medical Systems, Pao Alto, CA, USA) and the planning system Eclipse.

The study protocol was reviewed and approved by our local institutional review board and informed consent was given by all participants.

### 2.2. Patients

The study population consisted of 18 females and 28 males with a mean age of 57 years (range 19–87 years) at diagnosis (see Table 1 for patients characteristics). Squamous cell carcinoma was the most common histological type 39.1%, followed by anaplastic carcinoma (21.7%) and adenocarcinoma (8.7%). Most patients had an advanced stage disease, with 24 patients in stage 3 (52.2%) and 13 patients in stage 4 (28.3%). In contrast, only eight patients had stage 1 or 2 (17.4%).

### 2.3. Pre-Treatment Assessment

All patients required a complete medical history, physical examination and laboratory evaluation. Each individual case was discussed in the interdisciplinary tumor board. Systemic staging was performed according to the classification and staging system for nasopharyngeal cancer (AJCC-7th and AJCC-8th) [10,11]. Diagnostic contrast-enhanced MRI in the treatment position was performed and fused with the planning CT. The findings of the fiberoptic nasopharyngoscopy were taken into account and for selected cases, additional information from PET/CT images were used. The volume of the inner ear included the bony labyrinth and was defined individually on axial CT images. A senior physician in radiation oncology approved all contours [12].

### 2.4. Treatment

Almost all patients were treated with IMRT techniques (sliding window technique or volumetric modulated arc therapy) (97.8%). Only one patient received three-dimensional conventional radiotherapy. The clinical target volumes were contoured according to the International guideline for the delineation of the clinical target volumes (CTV) for nasopharyngeal carcinoma since 2017 [13]. Previously, the DAHANCA, EORTC, HKNPCSG, NCIC CTG, NCRI, RTOG, TROG consensus guidelines were used [14]. In particular, 4 risk CTVs (2 for primarius, 2 for LNs) were contured:

– CTVp 1 (= high-risk primary tumor − full therapeutic dose, 70–72 Gy) = GTV + 5 mm (+1 mm if tumor in close proximity to critical OARs).

– CTVp2 (= intermediate risk − prophylactic dose, 54–60 Gy) = CTVp1 + 5 mm.

– CTVn1 (= high-risk nodal volumes − full therapeutic dose, 70–72 Gy) = affected LN + 5 mm (possibly + 10 mm for ECE).

– CTVn2 (= intermediate risk nodal volumes − prophylactic dose, 54–60 Gy) = CTVn1 + 5 mm, but always includes Level II, III and Va.

Cisplatin, carboplatin, paclitaxel and 5-flourouracil were utilized as chemotherapeutic agents. In one case, immunomodulatory therapy was administered. In the case of impaired hearing before treatment, cisplatin was replaced by carboplatin/paclitaxel.

### 2.5. Follow-Up

All Patients were examined during regular radiotherapeutic and otorhinolaryngologist follow-up two months after RT and every 3–6 months afterwards using the NCI Common Terminology Criteria for Adverse Events (version 5). For hearing impairment and tinnitus the pre- and post-therapeutic audiograms of each ear were compared. If no audiogram was available, the clinical classification for adults was used [15].

### 2.6. NTCP Calculations

To perform an explaratory analysis concerning the relation between the administered radiation dose and the occurrence of inner ear toxicity, data from 38 patients were included. The maximum and mean radiation dose in Gy measured in the inner ear were extrapolated from the planning system. Doses to each ear were registered separately due to the possibility of asymmetric radiation exposure. For each data point, it was indicated whether it led to side effects or not.

For processing, the data points were grouped into bins, each consisting of a five gray interval. For each bin, the relative number of positive points to the total number of points in the bin was computed. Afterwards, the relative value was plotted over the corresponding radiation dose. As shown in the prospective study by Pan [9], an increase of side effects with increasing dose was expected. It was assumed that this increase is not linear, but increases sharply beyond a certain dose threshold, suggesting a sigmoid function. This sigmodial relationship enables application of an NTCP (Normal Tissue Complication Probability) model. To define this cut-off dose value as a dose constraint, the Cutoff Dose Logistic Regression Model (CDLR) was used, see Equation (1) [16].

As described by Burman et al. [7], there is a volume dependence of the complication probability for most organs. In small organs, no relevant differences can be found between distinctive partial volumes of the organ regarding the resulting probability of complications. This was shown by Burman et al. [7] for the middle/external ear, but may be also adapted to the inner ear, due to the comparable size.

(1) $$NTCP\left(D\right)=\frac{1}{\left(1+\exp(-D\times \beta +\gamma )\right)}$$

In this model *D* signifies the dose. To obtain the required regression parameters (β and γ), the weighted least squares method was used to fit the sigmoid to the data points. The weighting was done according to the total number of data points of the bin. As in Burman et al. [7] the determined *m* value describes the slope of the sigmoid function at a complication probability of 50% ($TD_{50}$). This was calculated numerically by the derivative at the point $TD_{50}$.

### 2.7. Statistical Analysis

Secondary endpoints of this study were overall survival (OS), progression free survival (PFS), locoregional control (LRC) and toxicity other than ototoxicity. OS was determined as the time from treatment initiation until death or loss to follow-up. PFS was defined as the duration from treatment initiation to any kind of disease recurrence after the radiotherapy treatment investigated in the study. LRC describes the time to locoregional recurrence. To estimate the LRC, PFS and OS the Kaplan-Meier method was utilized [17].

The statistical analysis was performed with IBM SPSS Statistics, version 28 (IBM, Armonk, NY, USA), and MATLAB, version R2020b (The MathWorks, Natick, MA, USA).

## 3. Results

Overall, 31 patients (67.4%) were treated with definitive radiotherapy, whereas 15 patients (32.6%) underwent postoperative radiotherapy. EBV-virus was detected in the histological examination of 15 patients (32.6%).

In total, 80.4% of the study population was treated with chemotherapy. Most patients received concurrent chemotherapy (78.3%), succeeded by adjuvant chemotherapy in 26.1% of cases. Only one patient received induction chemotherapy (2.2%). No chemotherapy was included in the treatment regimen of 9 patients (19.6%).

For concurrent chemotherapy, cisplatin 100mgm2 (n=17; 37.0%) for two or three cycles or 40mgm2 (n=12; 26.1%) for five or six cycles was most commonly administered. A few patients received carboplatin (AUC5) alone (n=3; 6.5%) or in combination with paclitaxel 40mgm2 (n=2; 4.3%). One patient was treated with one cycle of cisplatin and then switched to carboplatin for two cycles due to inner ear side effects. Another patient received cisplatin in combination with 5-flourouracil for two cycles and afterwards cisplatin alone for one cycle.

The most commonly applied adjuvant chemotherapy included cisplatin 80mgm2, with one patient receiving only 50mgm2, in combination with 5-flourouracil 1000mgm2 for three cycles (n=9; 19.6%). Again, in two patients who initially received cisplatin and 5-flourouracil cisplatin had to be changed to carboplatin. One patient was treated upfront with a combination of carboplatin and 5-flourouracil. One patient underwent prior chemotherapy with CHOP-14 due to malignant lymphoma. One patient in the recurrent situation received nivolumab 240 mg intravenously every 14 days.

### 3.1. Outcomes

**Figure 1:**
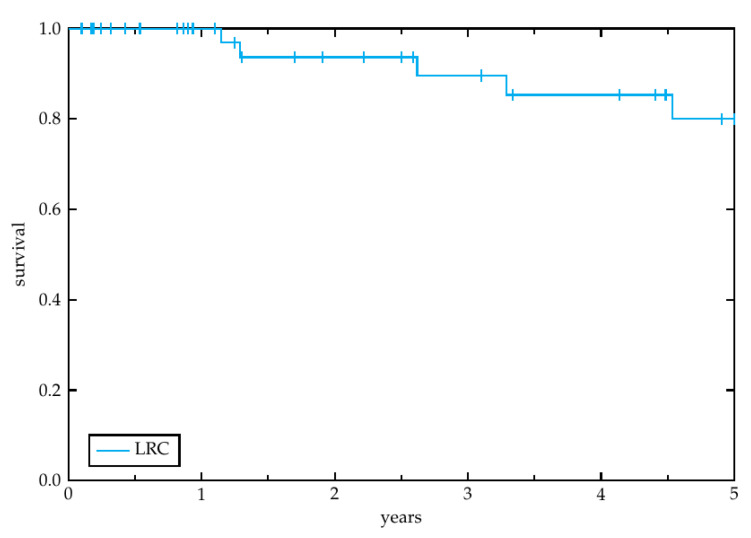
Locoregional control. Kaplan-Meier plot showing locoregional control of the 46 patients, enrolled in this study, within a 5-year period after radiotherapy initiation.

**Figure 2:**
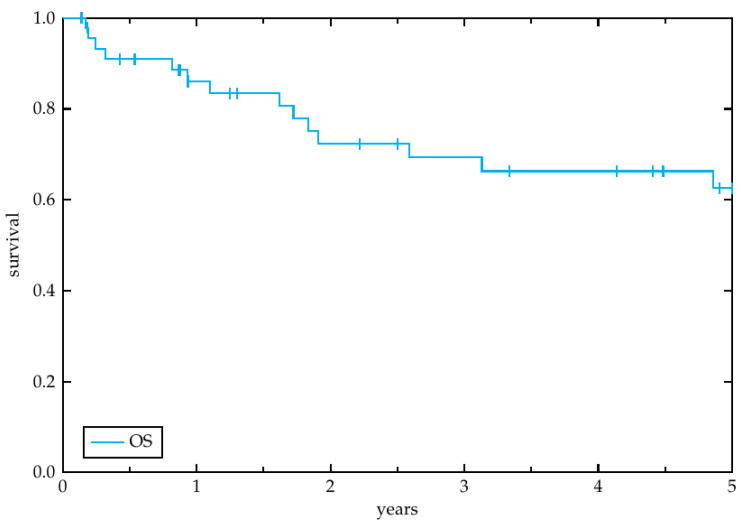
Overall survival. Kaplan-Meier plot representing the overall survival of the 46 patients, enrolled in this study, within the 5-year period after radiotherapy initiation.

**Figure 3:**
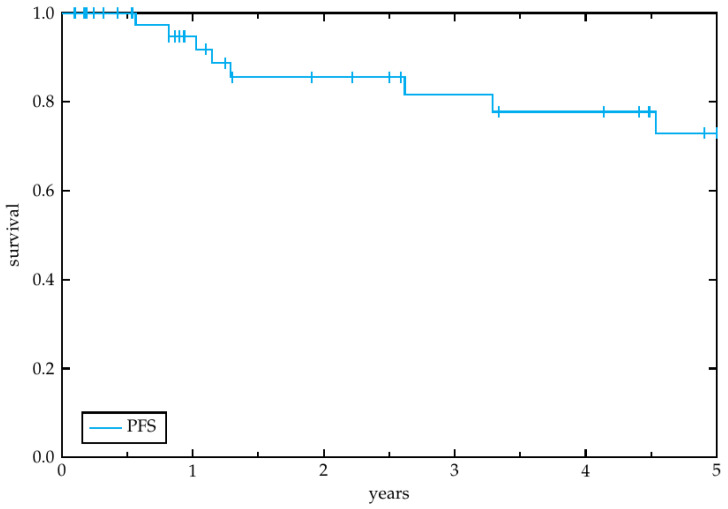
Progression free survival. Kaplan-Meier plot showing the progression free survival of the 46 patients, enrolled in this study, within the 5-year peroid after radiotherapy initiation.

The median follow-up time was 31.2 months (range 2.4–141.6 months). Overall, 11 patients (23.1%) experienced a recurrence and 19 patients (41.3%) died during the study period. Among the 11 patients with relapse, 7 had a locoregional recurrence (15.2%) and 4 had a distant recurrence (8.7%). Four patients had to interrupt radiotherapy due to decrease of performance status, grade 2–3 radiodermatitis, suspected retroperitoneal abscess, and febrile infection, respectively. One patient stopped therapy at our department voluntarily to continue radiotherapy at another institution. One patient stopped therapy against medical advice due to persistent decrease of performance status. The median overall survival amounted to 2.9 years (range 0.1–14.0 years). The estimated 3-year LRC (Figure 1), OS (Figure 2) and PFS (Figure 3) rates by the Kaplan-Meier method were 89.6%, 69.3% and 81.7%, respectively. The 5-year rates were 80.0%, 62.6% and 72.9%, respectively.

### 3.2. Toxicities

Treatment related toxicities are described in Table 2, Table 3 and Table 4.

**Figure 4:**
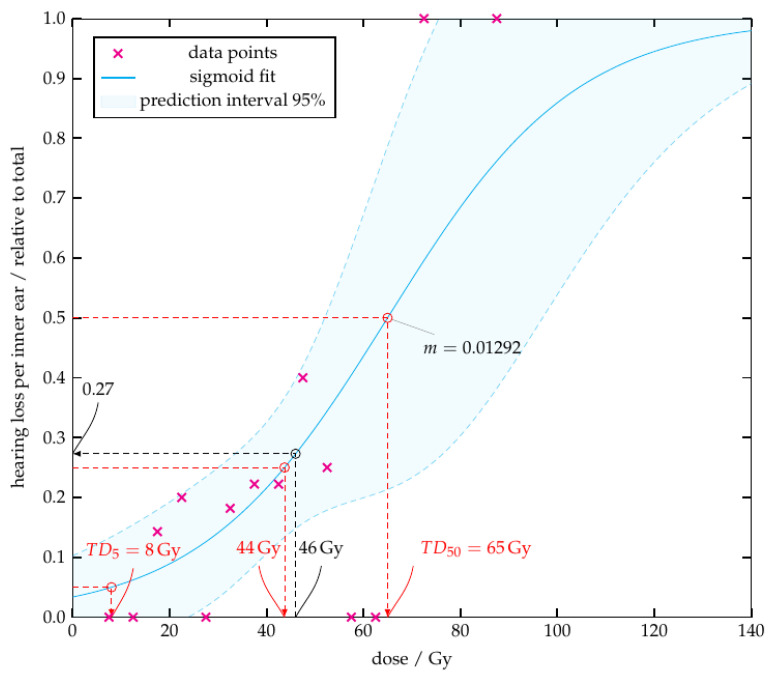
Inner ear side effects depending on mean radiation dose. The curve describes the incidence of inner ear side effects depending on the dose administered to the inner ear.

**Figure 5:**
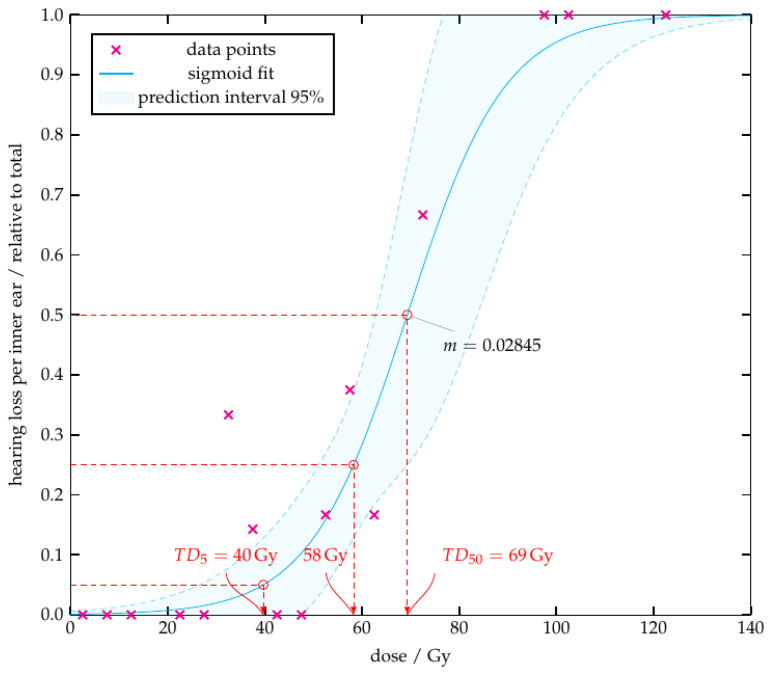
Inner ear side effects depending on maximum radiation dose. The curve describes the incidence of inner ear side effects depending on the dose administered to the inner ear.

During course of radiotherapy and follow-up, in 11 cases (23.9%) the inner ear was affected by side effects (tinnitus: n=4, hearing impairment n=7). As shown in Figure 4 and Figure 5, an increase of radiation dose administered to the inner ear leads to an increased rate of inner ear side effects. A sigmoid function could be modelled successfully and the estimated parameters can be found in Table 5.

With a mean radiation dose of 44Gy per inner ear, 25% of patients would experience inner ear side effects, whereas at a radiation dose of 65Gy, 50% of patients would be affected. The maximum slope (*m*) at 50% is m=0.013. Considering the maximum radiation dose, the dose for the complication probability of 25% and 50% would be 58Gy and 69Gy, respectively. The maximum slope at 50% is m=0.025.

## 4. Discussion

This 17-year single institution study presents an NTCP model for the inner ear, based on clinical data in the setting of IMRT treatment. We were able to verify a sigmoidal relation between radiation dose and incidence of inner ear toxicities tinnitus and/or hearing impairment and derived dose constraints. A Dmean <44Gy or <65Gy has to be respected to limit the probability of inner ear toxicity <25% ($TD_{25}$) or <50% ($TD_{50}$), respectively, with the Dmax being 58Gy ($TD_{25}$) and 69Gy ($TD_{50}$). Therefore, these constraints may serve as anchor points for radiation plan evaluation and should be maintained, if compatible with target volumes.

**Figure 6:**
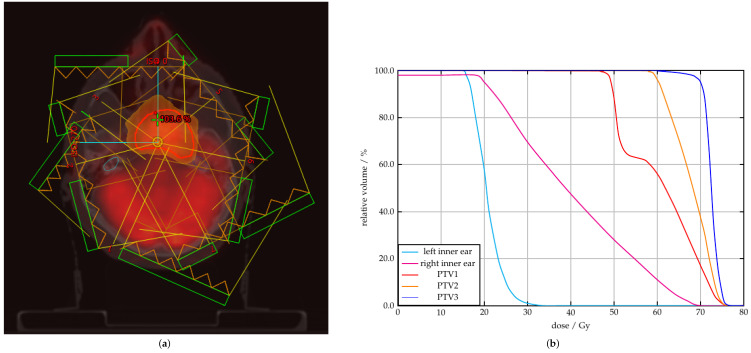
Dose distribution and dose volume histogram of definitive radiochemotherapy of nasopharyngeal cancer with clivus infiltration. (**a**) Tumor in the left paramedian nasopharynx, exceeding the midline, with high glucose metabolism, SUV max 16.5 and arrosion of the clivus on the left side adjacent to the left inner ear. 95% Isodose of the radiotherapy plan with protection of the inner ears using intensity modulated radiotherapy in 7-field sliding-window technique. Nevertheless, especially the left inner ear is exposed to high doses and unfortunately grade 3 hearing impairment occurred. (**b**) Corresponding cumulative dose-volume histogram: PTV1: Bilateral regional lymphatic drainage pathways and nasopharyngeal area with 50.4 Gy PTV2: Affected lymphatic drainage pathways and primary tumor with 59.4 Gy PTV3: Nasopharynx tumor and the affected lymph nodes on both sides with 72.0 Gy Right inner ear: Dmax 33.6; Dmean 20.8 Gy Left inner ear: Dmax 70.5 Gy; Dmean 39.9 Gy.

These data may be used as a guidance to counsel patients accordingly and to adapt radiation plans. Nevertheless, for patients with invasion to the clivus, pterygopalatine fossa, cavernous sinus, sphenoid sinus, the inner dose may not be easily lowered, as high doses are needed to achieve optimal levels of tumor control (Figure 6).

In the literature, Burman et al. investigated the relation between radiation dose and complication probability using the NTCP model. For the middle and external ear they found a $TD_{25}$ of 40Gy and 65Gy for acute serous otitis and chronic serous otitis, respectively. However, they did not investigate the inner ear, which could possibly be a more important risk organ, since severe late toxicities such as tinnitus, hearing impairment and loss possibly may occur [7].

To date, only a few studies have investigated the occurrence of side effects in the inner ear in relation to the radiation dose. Among them, Lee et al. analyzed the impact of IMRT in patients with head-and-neck cancer on the cochlea (NTCP-fitted parameters $TD_{50}=46.31\mathrm{Gy}$ and for the logistic and LKB models $TD_{50}=46.52\mathrm{Gy}$). A mean dose for the cochlea <32Gy is recommended to limit the probability of tinnitus <20% [8].

The study of Chen et al. evaluated the dose-response relationship of radiation dose and sensorineural hearing loss. According to this study, a mean dose of 48Gy should not be exceeded, with a rapidly increasing incidence of sensorineural hearing beyond this value [18].

In the retrospective analysis by Bhandare et al., a distinct increase in the incidence of sensorineural hearing loss from a total dose >55Gy to the cochlea was shown. For all components of the auditory system, an increasing incidence of ototoxicity was observed between 60–66Gy [19].

In the study by Espenel et al., dose constraints of 10Gy for radiochemotherapy and 40Gy for radiotherapy alone were proposed. In addition, monitoring of ototoxicity during treatment is recommended to allow timely intervention [20].

In other studies, values between 45–50Gy were described as dose constraints for the mean radiation dose [9,21,22]. In the current study, this would correspond to an incidence of inner ear side effects of 26–31.5%, as shown in Figure 4. In order to maintain a similar risk level compared to the literature, we accepted a complication probability of 25%, which resulting in a mean radiation dose of 44Gy.

However, inner ear damage is a multifactorial process, the incidence of which may be modulated by damage from previous otitis media, chemotherapeutic agents and intracranial recurrences in addition to radiation [23]. Regarding chemotherapy, especially cisplatin is recognized as an ototoxic agent [6]. Wei et al. showed that patients treated with concurrent or adjuvant chemotherapy in addition to radiotherapy for nasopharyngeal carcinoma had more severe inner ear damage in comparison to patients undergoing radiotherapy as a monotherapy [5]. Correspondingly, the analysis by Bhandare et al. suggested that combined radiochemotherapy requires lower radiation doses to the cochlea till damage than radiotherapy alone [24].

Modern radiation techniques like IMRT technique are known to reduce acute and late side effects such as xerostomia, therby also improving the quality of life [3,25,26,27,28]. In particular, IMRT allows better sparing of the inner ear reducing radiation-induced ototoxicity [29].

In a retrospective study, Liu et al. demonstrated an approach to reduce damage to the inner ear and the parotid gland by reducing the clinical target volume (CTV). For this purpose, patients who received conventional-volume IMRT and patients who received reduced-volume IMRT were matched and compared. Decreased CTV did not significantly affect survival, but resulted in reduced late side effects such as xerostomia (p=0.04) and hearing loss (p=0.01). Particular patients with T1–2 stage, N0–1 stage and Stage 1–2 were described as good candidates for this approach. This strategy is enabled by modern techniques like IMRT [30].

A few studies investigated cochlea-sparing radiation planning to reduce ototoxicity. In the plan-study by Lamaj et al., irradiation plans were optimized retrospectively to reduce the dose for both cochlea without affecting PTV and other values. A mean dose of 14.97Gy (left) and 18.47Gy (right) could be achieved with VMAT which was significantly lower as in the previous plans with 24.09Gy (left) and 26.05Gy (right) [31].

In the study by Braun et al., a unilateral cochlea-sparing optimization of radiation plans was performed. A mean dose of <10Gy was achieved in all radiation plans. The mean cochlear dose was 6.8Gy (patients with definitive RCT) and 7.6Gy (patients with adjuvant RCT), respectively, in contrast to the contralateral, non-spared cochlea with 18.5Gy and 29.8Gy, respectively [32].

This study has limitations, since both chemo- and radiotherapy are ototoxic which makes attribution of side-effects to one treatment modality ambiguous in many cases. Patient number and therefore data points are limited, which is accompanied by an uncertainty regarding the definition of dose contraints. As a monocentric and retrospective analysis, data are sometimes incomplete also affecting risk rates and model accuracy. In addition, the entire inner ear was contoured rather than the cochlea separately.

The proposed NTCP models enables the derivation of individual dose constraints depending on the accepted propability for inner ear toxicities. These data may be used as a guidance to counsel patients accordingly and to adapt radiation plans with excessive long-term risks. Multi-institutional analysis and big-data assessments via artificial intelligence may help to further deepen the understanding and ameliorate the predicition of this important side-effect in the future.

## Figures and Tables

**Table 1:** Characteristics of the study population. * One patient had an angiocentric nasal T-cell lymphoma stage II-IIIb.

Age Range 19–87 Years / Mean Age 57 Years / ($n_{\mathrm{total}}=46$)		
**Characteristics**	**Affected Patients**	**Percentage %**
**Sex**		
Female	18	39.1
Male	28	60.9
**Primary location**		
Nasopharynx	41	89.1
Naso-/Oropharynx	5	10.9
**Histology**		
Squamous cell carcinoma	18	39.1
Anaplastic carcinoma	10	21.7
Adenocarcinoma	4	8.7
Adenoid cystic carcinoma	3	6.5
Neuroendocrine carcinoma	2	4.3
Transitional cell carcinoma	2	4.3
other	2	4.3
unknown	5	10.9
**Grading**		
Grade 1	1	2.2
Grade 2	7	15.2
Grade 3	15	32.6
Grade 4	12	26.1
unknown	11	23.9
TNM-Classification *		
T stage 1	9	19.6
2	11	23.9
3	15	32.6
4	10	21.7
N stage 1	9	19.6
2	9	19.6
3	5	10.9
M stage 0	43	93.5
1	2	4.3
Staging *		
Stage 1	2	4.3
Stage 2	6	13.0
Stage 3	24	52.2
Stage 4a	11	23.9
Stage 4b	2	4.3
**Recurrence**		
none	22	47.8
locoregional	7	15.2
distant	4	8.7
unknown	13	28.3
**Radiotherapy technique**		
IMRT	45	97.8
3D-CRT	1	2.2
**Chemotherapy**		
prior	1	2.2
concurrent	36	78.3
adjuvant	12	26.1

**Table 2:** Acute side effect of chemotherapy and its proportion in the total population and proportion in the chemotherapy subpopulation.

Type	Occurring Cases	Cases in % ($n_{\mathrm{total}}=46$)	Cases in % ($n_{\mathrm{chemotherapy}}=37$)
Leukopenia	15	32.6	40.5
Nausea/emesis	9	19.6	24.3
Dysphagia/ odynophagia	8	17.4	21.6
Decrease of performance status	4	8.7	10.8
Inner ear side effects	4	8.7	10.8
Mucositis	3	6.5	8.1
Oral candidiasis	2	4.3	5.4
Fatigue	2	4.3	5.4
Polyneuropathy	2	4.3	5.4

**Table 3:** Acute side effects of radiotherapy, classified by type and grade.

Type	Occurring Cases	Grade 1	Grade 2	Grade 3	Unknown Grade
Dermatitis	30 (65.2%)	7 (15.2%)	12 (26.1%)	7 (15.2%)	4 (8.7%)
Mucositis	24 (52.2%)	6 (13.0%)	3 (6.5%)	6 (13.0%)	9 (19.6%)

**Table 4:** Late side effects of radiotherapy, classified by type and grade.

Type	Occurring Cases	Grade 1	Grade 2	Grade 3	Unknown Grade
Xerostomia	29 (63.0%)	-	-	-	-
Dysgeusia	20 (43.5%)	17 (37.0%)	3 (6.5%)	-	-
Dysphagia	16 (34.8%)	6 (13.3%)	4 (8.7%)	1 (2.2%)	5 (10.9%)
Hearing impairment	7 (15.2%)	2 (4.3%)	2 (4.3%)	3 (6.5%)	-
Tinnitus	4 (8.7%)	4 (8.7%)	-	-	-
Fatigue	8 (17.4%)	-	-	-	-
Dysphonia	6 (13.0%)	-	-	-	-

**Table 5:** Estimated regression parameters.

Type	β	γ	*m*
mean	0.0517	3.3587	0.01292
max	0.0994	6.8876	0.02845

## Data Availability

Original data will be provided upon request.

## Abbreviations

The following abbreviations are used in this manuscript:

CDLR	Cutoff dose logistic regression model
CT	Computed tomography
CTV	Clinical target volume
EBV	Epstein-Barr virus
ECE	Extracapsular extension
GTV	Gross tumor volume
Gy	Gray
IMRT	Intensity-modulated radiotherapy
LN	lymph node
LRC	Locoregional control
MRI	Magnetic resonance imaging
NPC	Nasopharyngeal carcinoma
NTCP	Normal tissue complication rate
OAR	Organs at risk
OS	Overall survival
PET	Positron emission tomography
PFS	Progression free survival
PTV	Planning target volume
RCT	Radiochemotherapy
RT	Radiotherapy
VMAT	Volumetric intensity modulated arc therapy
